# Few generalized entropic relations related to Rydberg atoms

**DOI:** 10.1038/s41598-022-10854-0

**Published:** 2022-05-06

**Authors:** Kirtee Kumar, Vinod Prasad

**Affiliations:** 1 Department of Physics and Astrophysics, University of Delhi, Delhi, 110007 India; 2 Department of Physics, Motilal Nehru College, University of Delhi, Delhi, 110021 India; 3 Department of Physics, Swami Shraddhanand College, University of Delhi, Delhi, 110036 India

**Keywords:** Atomic and molecular physics, Information theory and computation

## Abstract

We calculate the analytical and numerical values of the position space Shannon entropy, momentum space Shannon entropy, and total Shannon entropy, $S_\rho$, $S_\gamma$, and $S_T$, respectively, of free and trapped Rydberg hydrogen-like atoms. The influence of atomic number *Z*, the principal quantum number *n*, and energy *E* on the Shannon entropy of the Rydberg atoms are illustrated. The scaling properties of Shannon entropy with energy of states *E* and the principal quantum number *n* have been reported for the first time to the best of our knowledge. Our work explains how Shannon entropy indicates localization-delocalization of the wavefunction. The total Shannon entropy as a measure of the number of nodes in the trapped Rydberg atom’s wavefunction is also discussed. We show why an uncertainty relation based on Shannon entropy is superior to Heisenberg uncertainty for Rydberg atoms.

## Introduction

Rydberg atoms are atoms in which one or more valence electron can be excited in states with extremely high principal quantum numbers *n*^1,2^. The valence electron is predominantly affected in such an atom by the positive charge of the ionic centre, not by its composition. These atoms that demonstrate the consistency of thought between the world of classical mechanics and quantum mechanics are important to study the correlation of classical and quantum regime. Although the study of Rydberg atoms has a long history, the advancement of laser technology has led to great experimental advances for researchers and has revived interest in such studies. Rydberg atoms are also significant in many research studies of astrophysics. These states in theory, give some advantages that could be exploited in the research for new applications. The special properties of Rydberg atoms, *i*.*e*., their extreme polarizability, long-range interaction, and long lifetime, have positioned them at the centres of highly active research areas of modern atomic physics and quantum information technology. In 2000, Jaksch et al. proposed a method of generating a fast phase gate using Rydberg atoms, which was the first proposal to use the blockade for quantum information^3^. It was further extended to a mesoscopic regime of many-atom ensemble qubits^4^. Rydberg blockade and antiblockade has also been suggested as a way to generate many-particle entanglement^5–12^. Carr and Saffman have proposed and analysed an approach for preparation of high fidelity entanglement and anti-ferromagnetic states using Rydberg mediated interaction with dissipation for two atom singlet^13^. It was further extended to a stationary three-dimensional entanglement between two-individual neutral Rydberg atoms^14^ and maximally entangled states via dissipative Rydberg pumping^15^. In the excitation spectra of ultra cold atoms of Rubidium and Cesium in their Rydberg states, dipole matrix elements and relevant energies were calculated using quantum defect theory^16^. So, there are many studies related to Rydberg atoms which are promising platform for quantum state engineering, quantum metrology, quantum simulation^17,18^, quantum information^21^, quantum computing^19,20,22^, sensing and imaging and quantum optics^23^.

Firstly, Michels et al. introduced the idea of study of confined atom. They studied spectral broading of the hydrogen atom inside an impenetrable spherical cavity^24^. The trapped atoms show enhanced response to external perturbation compare to free atoms. In recently, mostly experiments are performed with Rydberg atoms in optical dipole traps and arrays of optical dipole traps. The atoms are temporarily excited to Rydberg states using resonant laser radiation. Typically, the ground-state atoms are trapped by off-resonant trapping radiation, but Rydberg atoms are not trapped. Rydberg atoms are repelled by the Laser-induced ponderomotive potential^25^ and transversely trapped in the light tube for times up to 10 *ms*. The experiments of trapping Rydberg atoms have been reported in recent years^26–32^. The theoretical description of trapped Rydberg atoms and their interaction with light and with each other is commonly performed using conventional quantum mechanical approach based on Schrodinger equation. The trapped Rydberg atoms have more quantum information than the free Rydberg atoms because the free atoms interact only at very short range^33^. The trapped Rydberg atoms open up new possibilities for applications of quantum optics and quantum information^34,35^. Therefore, trapped Rydberg atoms have taken importance in recent studies^36^.

The spread of the probability densities in both position and momentum space determine the physical and chemical properties of the Rydberg atoms. The multiple facets of this spread are currently quantified not only by means of radial expectation values in position and momentum spaces, but also by certain local and global information-theoretical measures. Various fundamental and/or experimentally measurable quantities such as the diamagnetic susceptibility, the potential energy, the kinetic energy, etc. are closely related to these spreading measures. In addition, they allow to determine various uncertainty measures.

There has been continuous interest in the studies on information theoretic measures for quantum mechanical systems. Entropy is a measure of the uncertainty associated with a random variable in information theory. Entropy usually refers to the Shannon entropy in this field, which measures the expected value of the information stored in a message, usually in units such as bits, i.e., when the value of the random variable is unknown, it is a measure of the average information content that is missing. Claude E. Shannon presented this idea in his paper “A Mathematical Theory of Communication” in 1948, in which he set out to find fundamental limits on signal processing operations such as data compression and data storage and communication reliability^37^. In other fields, such as statistical mechanics, cryptography, quantum computing^38^, atomic and molecular physics^39–45^ and chemistry^46,47^, this theory has bee extended to a range of applications since its proposal.

From the underlying concepts of information theory, the global measure of Shannon entropy is fundamental to quantum information-theoretical measures.There are other global measures, besides Shannon entropy, which include Tsallis and Renyi entropies and Onicescu energy^48–51^. The importance of the global measure is to study the uncertainty associated with the distribution of probability^52–55^. An uncertainty relation based on the Shannon entropy known as BBM inequality derived by Beckner-Bialynicki-Birula and Mycieslki^56,57^ which is a stronger version of the Heisenberg uncertainty principle of quantum mechanics, which is written as

1 $$\begin{aligned} S_T = S_{\rho } + S_{\gamma }\ge & {} \,\,d(1+\ln \pi ) \end{aligned}$$

where, *d* is the spatial dimension, $S_{\rho }$ and $S_{\gamma }$ are Shannon entropy for position and momentum spaces, which are expressed as

2 $$S_{\rho } = - \int {\rho \left( {\mathop{r}\limits^\rightarrow } \right)\ln \rho \left( {\mathop{r}\limits^\rightarrow } \right)} {{d}}\mathop{r}\limits^\rightarrow$$

3 $$S_{\gamma } = {\text{ }} - \int {\gamma \left( {\mathop{p}\limits^\rightarrow } \right)\ln \gamma \left( {\mathop{p}\limits^\rightarrow } \right)} d\mathop{p}\limits^\rightarrow$$

respectively. Where, $\rho ({\mathop{r}\limits^\rightarrow })$ and $\gamma ({\mathop{p}\limits^\rightarrow })$ are the radial probability density in position and momentum space respectively and they are written as $\rho ({\mathop{r}\limits^\rightarrow }) = |\Psi ({\mathop{r}\limits^\rightarrow })|^2$ and $\gamma ({\mathop{p}\limits^\rightarrow }) = |\Phi ({\mathop{p}\limits^\rightarrow })|^2$, where $\Psi ({\mathop{r}\limits^\rightarrow })$ and $\Phi ({\mathop{p}\limits^\rightarrow })$ are the normalized wave function in position and momentum space respectively. One of the implications of the BBM inequality is that the lower bound values of the Shannon entropy sum are interpreted in such a way that if the position entropy increases, the momentum entropy will decrease in such a way that its sum follows the bound of BBM inequality. In physical science, Shannon entropy is a hypothesis that describes the spatial distribution of the wave function for different states. The concentration of wave function of the state is higher when the Shannon entropy is small^58^. We may also assume that the wavefunction is localized when the Shannon entropy is low and delocalized when it is high. As a consequence, Shannon entropy can be used to estimate the stability of a system. When Shannon entropy is low, it is likely to be more stable, and when Shannon entropy is high, it is assumed to be unstable. Shannon entropy is also important in the study of the structure and dynamics of atomic and molecular systems since it is related to fundamental and experimentally observable quantities like kinetic energy and magnetic susceptibility^59^. Shannon entropy has attracted a lot of attention due to its application in different fields. Saha and Jose use Shannon entropy as an indicator of correlation and relativistic effects in confined atoms^60^. Recently, many researchers have used various potentials to study the Shannon entropy^61–64^. Shannon entropy for quantum heterostructures has also been studied^65–67^. The Shannon entropy of the confined hydrogenic atoms have been calculated in many previous works^68–71^. Very recently, there are studies on the influence of electric field on the Shannon entropy^72,73^. As a result, we intend to investigate the analytical and numerical calculation of Shannon entropy and various entropic uncertainty relations^74–78^ for the Rydberg hydrogen atom, as suggested in the work’s concept.

## Method

The non-relativistic Hamiltonian of a Hydrogen-like atom of mass *M* and atomic number *Z* with the nucleus placed in the center of an impenetrable sphere is

4 $$\begin{aligned} H= & {} \frac{p^2}{2M} - \frac{Ze^2}{r} + V_{c}(r) \end{aligned}$$

where *e* is the charge of electron and $V_{c}(r)$ is the spherical hard confining potential which is written as

5 $$\begin{aligned} V_{c}(r)= & {} \left\{ \begin{array}{rcl} 0 &{} \text{ for } &{} r\le r_{0}\\ \infty &{} \text{ for } &{} r>r_{0} \end{array}\right. \end{aligned}$$

Where $r_{0}$ is the radius of spherical confinement.

Using $p = -\iota \hbar \nabla$, Hamiltonian becomes

6 $$\begin{aligned} H= & {} \frac{-\hbar ^2\nabla ^2}{2M} - \frac{Ze^2}{r} + V_{c}(r) \end{aligned}$$

Using atomic unit (a.u.) ($\hbar$ = 1, *M* = 1 and *e* = 1), Hamiltonian can be written as

7 $$\begin{aligned} H= & {} \frac{-\nabla ^2}{2} - \frac{Z}{r} + V_{c}(r) \end{aligned}$$

So, the Schrodinger equation $H\Psi = \,\,E\Psi$ is written as

8 $$\begin{aligned} (\frac{-1}{2}\nabla ^2 - \frac{Z}{r})\Psi= \,& {} \,\,\,E\Psi \end{aligned}$$

In spherical coordinates the Schrodinger equation is written as

9 $$\begin{aligned} \frac{-1}{2}\left[ \begin{array}{cc|r}\frac{1}{r^2}\frac{\partial }{\partial r}(r^2\frac{\partial \Psi }{\partial r}) + \frac{1}{r^2 \sin \theta }\frac{\partial }{\partial \theta }(\sin \theta \frac{\partial \Psi }{\partial \theta }) + \frac{1}{r^2 \sin ^2\theta }(\frac{\partial ^2\Psi }{\partial \phi ^2}) \end{array} \right] - \frac{Z}{r}\Psi = E\Psi \end{aligned}$$

Because of spherical symmetry the above Schrodinger equation can be solved by the method of separation of variables. So, complete normalized wavefunction $\Psi _{nlm}(r,\theta ,\phi )$ can be written as

10 $$\begin{aligned} \Psi _{nlm}(r,\theta ,\phi )= \,& {} \,\,\,R_{nl}(r)Y_{lm}(\theta ,\phi ) \end{aligned}$$

where, $R_{nl}(r)$ represents the radial part and $Y_{lm}(\theta ,\phi )$ is the angular part of the wavefunction. The angular part of wavefunction $Y_{lm}(\theta ,\phi )$ is same and radial part $R_{nl}(r)$ is affected due to confining potential. So, the radial Schrodinger equation can be written as

11 $$\begin{aligned} \begin{aligned} \frac{d}{dr}\left( \begin{array}{cc} r^2 \frac{dR_{nl}(r)}{dr}\end{array} \right) -2r^2\left[ \begin{array}{cc|r} - \frac{Z}{r}+\frac{l(l+1)}{2r^2}-E\end{array} \right] R_{nl}(r)=0 \end{aligned} \end{aligned}$$

Using $R_{nl}(r) = \frac{\xi _{nl}(r)}{r}$, the radial equation finally reduces to the following form

12 $$\begin{aligned} \frac{-1}{2}\frac{d^2\xi }{dr^2}+V_{eff}\xi =E\xi \end{aligned}$$

where,$V_{eff}$ is effective potential which is defined as $V_{eff} = - \frac{Z}{r}+\frac{l(l+1)}{2r^2}$.

For given effective potential, Eq. (12) is solved numerically by 9th order finite difference method using MATLAB. The eigenvalues equation for radial Schrodinger equation is reduced to a matrix form. Diagnoalizing this matrix the eigenvalues and eigenvectors are obtained. So, radial wavefunctions $R_{nl}(r)$ are obtained in position space using these eigenvectors. So, total normalized wavefunction $\Psi _{nlm}({\mathop{r}\limits^\rightarrow }) = R_{nl}(r)Y_{lm}(\theta ,\phi )$ can written in position space. Using Dirac-Fourier transformation, the momentum space wavefunction of the position space wavefunction can be written as

13 $$\Phi \left( {\mathop{p}\limits^\rightarrow } \right) = {\text{ }}\frac{1}{{(2\pi )^{{\frac{3}{2}}} }}\int {e^{{ - \iota \mathop{p}\limits^\rightarrow .\mathop{r}\limits^\rightarrow }} \Psi \left( {\mathop{r}\limits^\rightarrow } \right)} d\mathop{r}\limits^\rightarrow$$

Using $\Psi ({\mathop{r}\limits^\rightarrow})$ and $\Phi ({\mathop{p}\limits^\rightarrow})$, we obtain probability density $\rho ({\mathop{r}\limits^\rightarrow}) = |\Psi ({\mathop{p}\limits^\rightarrow})|^2$ in position space and $\gamma ({\mathop{p}\limits^\rightarrow}) = |\Phi ({\mathop{p}\limits^\rightarrow})|^2$ in momentum space respectively. From $\rho ({\mathop{r}\limits^\rightarrow})$ and $\gamma ({\mathop{p}\limits^\rightarrow})$, we calculate the Shannon entropy $S_\rho$ in position space and the Shannon entropy in momentum space $S_\gamma$ respectively, which are presented in next section.

## Results and discussion

In the present work, we studied the differences in Shannon entropy between free Rydberg atoms and confined (trapped) Rydberg atoms. The study is limited to the *S* states with zero angular momentum (*l* = 0), which are known as linear states. As a model, we considered a spherical trapping potential with infinite wells. We have used atomic units throughout this paper.

### Free linear Rydberg states

**Figure 1:**
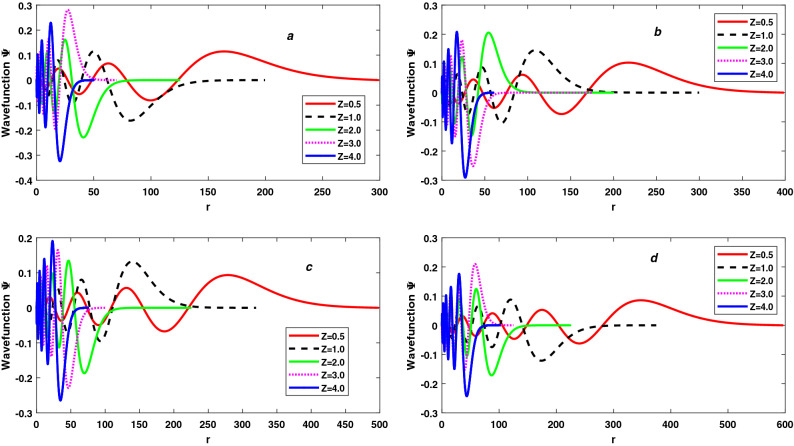
The variation of Wavefunction $\Psi$ with r and atomic number Z for 7s state (**a**), 8s state (**b**), 9s state (**c**) and 10s state (**d**) of free Hydrogen-like atoms.

In Table 1, we have presented the values of the Shannon entropy in position space $S_\rho$, the Shannon entropy in momentum space $S_\gamma$, Heisenberg uncertainty principle (HUP) and uncertainty relation based on the Shannon entropy ($S_T = S_\rho + S_\gamma$) with atomic number *Z* for $n \ge 6$ for s-states of Hydrogen-like atoms. We note that as the atomic number *Z* increases while the principal quantum number *n* remains constant, the energy becomes more negative, implying that the energy decreases as *Z* increases. The nucleus has a more positive charge as *Z* increases, so the coulombic attraction force between the nucleus and electron increases, causing the nucleus to hold the electron tighter. The energy becomes less negative as *n* is raised while holding *Z* unchanged, showing that energy increases as *n* is raised. It is because of increasing of *n*, the distance between the nucleus and electron increases so the coulombic attraction force between the nucleus and electron decreases, due to this nucleus holds the electron less tight. As *Z* increases while *n* remains constant, potential energy becomes more negative, meaning that potential energy decreases as kinetic energy rises. As *Z* increases, the wavefunction of the electron compresses in position space and expands in momentum space, resulting in a wavefunction that is localized in position space but delocalized in momentum space. However, we know that as *n* increases while *Z* remains constant, the distance between the nucleus and electron increases, therefore potential energy becomes less negative, meaning that potential energy increases and kinetic energy decreases. As *n* increases, the wavefunction of the electron expands in position space and contracts in momentum space, resulting in wavefunction delocalization in position space and localization in momentum space, as shown in Fig. 1. Standard deviation and Shannon entropy can also be used to explain this. With principal quantum number *n* and atomic number *Z*, we observed the variation of variance in position space $\Delta r$ and variance in momentum space $\Delta p$. Keeping *n* constant, $\Delta r$ decreases for increasing of *Z* and $\Delta p$ increases for increasing of *Z*. However, if *Z* remains unchanged, $\Delta r$ increases as *n* increases, and $\Delta p$ decreases as *n* increases. This also shows that the wavefunction in position space becomes localized and delocalized in momentum space as *Z* increases (while holding *n* constant), and vice versa. Here, we can also notice that product of variance $\Delta r\Delta p$ is independent with *Z* and equal for all values of *Z*, but it depends on *n* and increases for increasing of *n*. Now we can see how Shannon entropy changes with principal quantum number *n* and atomic number *Z* in linear Rydberg states. Rosa et al. derived and analyzed the expressions for entropy and complexity of linear Rydberg states of Hydrogenic atoms^79^. Yanez et al. obtained the analytical results for the $S_T$ of free H-like atoms^80,81^. The scaling properties of Shannon entropy with atomic number *Z* were also discussed in previous works^82,83^. Guevara et al.^84^ obtained $S_\rho$ and $S_\gamma$ as a function atomic number *Z* for the ground state of H-like atoms and have made an interesting observation that total Shannon entropy $S_T$ is independent of *Z* within the series and that for the ground state 1*s* the Shannon entropy for position space and momentum space are, respectively given by

14 $$\begin{aligned} S_\rho=\,& {} \ln \pi - 3\ln Z + 3 \end{aligned}$$

15 $$\begin{aligned} S_\gamma= & {} \,\,\,5\ln 2 + 2\ln \pi + 3\ln Z - \frac{10}{3} \end{aligned}$$

We use *n*s states position space wavefunction of the Hydrogen-like atom and obtain the corresponding momentum space wavefunction using Dirac-Fourier transformation (Eq. (13)) and obtain $S_\rho$ and $S_\gamma$ using Eq. (2) and (3) as a function of principal quantum number *n* and atomic number *Z* for free system, respectively can be defined as

16 $$\begin{aligned} S_\rho (n,Z) \approx \frac{23}{4}\ln n + \ln \pi - 3\ln Z + 3 \end{aligned}$$

17 $$\begin{aligned} S_\gamma (n,Z) \approx -\frac{15}{4}\ln n + 5\ln 2 + 2\ln \pi + 3\ln Z - \frac{10}{3} \end{aligned}$$

**Figure 2:**
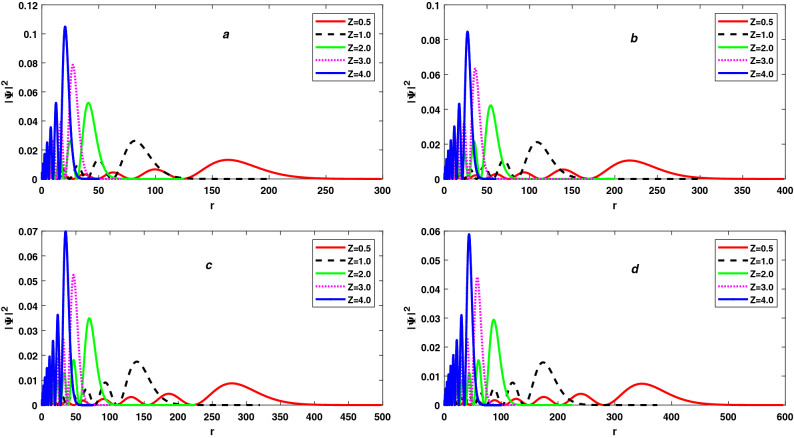
The variation of Probability density $|\Psi |^2$ with r and atomic number Z for 7s state (**a**), 8s state (**b**), 9s state (**c**) and 10s state (**d**) of free Hydrogen-like atoms.

Summing Eqs. (16) and (17) and we get total Shannon entropy

18 $$\begin{aligned} S_T \approx 2\ln n + 5\ln 2 +3\ln \pi - \frac{1}{3} \end{aligned}$$

which is not affected by the value of *Z*. Thus, in a one-electron atomic structure, $S_T$ is a function of the principal quantum number *n* rather than the atomic number *Z*. Now that we’ve gone over our numerical result from Table 1, we can see that $S_T$ remains constant as *Z* changes (while keeping principal quantum number *n* constant) and that $S_T$ increases as principal quantum number *n* increases.As a result, our results satisfy the *Z* and *n* dependency described by Eq. (18). We’ve found that $S_T$ follows the bound of the BBM inequality, which is defined by Eq. (1). Shannon entropy in position space $S_\rho$ and Shannon entropy in momentum space $S_\gamma$ numerical values satisfied the analytical values of $S_\rho$ (Eq. (16)) and $S_\gamma$ (Eq. (17)) for all values of *Z* and *n*. Shannon entropy in position space $S_\rho$ decreases while in momentum space $S_\gamma$ increases for increasing of *Z* holding *n* constant. But, $S_\rho$ increases for increasing of *n* and $S_\gamma$ decreases for increasing of *n* holding *Z* constant. Increasing Shannon entropy denotes wavefunction delocalization (wavefunction expansion), while decreasing Shannon entropy denotes wavefunction localization (wavefunction compression). As a conclusion of our results, we can state that the wavefunction in position space is localized while wavefunction in momentum space becomes delocalize as *Z* increases (while *n* remains constant), and vice versa as *n* increases (while *Z* remains constant). Figures 1 and 2 represent the variance of the wavefunction and electron probability density in position space as a function of *r* with *Z* and *n*, respectively, demonstrating that raising *Z* compresses both the wavefunction and the electron probability density while increasing *n* expands both.

**Table 1:** The variation of Energy E, variance in position space $\Delta r$, variance in momentum space $\Delta p$, Shannon entropy in position space $S_{\rho }$, Shannon entropy in momentum space $S_{\gamma }$, Heisenberg uncertainty principal $\Delta r \Delta p$ and uncertainty relation based on entropy $S_T=S_{\rho } + S_{\gamma }$ with atomic number *Z* and principal quantum number *n* for Rydberg linear states of free Hydrogen-like atoms.

States	Z	E	$\Delta r$	$\Delta p$	$S_{\rho }$	$S_{\gamma }$	$\Delta r \Delta p$	$S_T=S_{\rho } + S_{\gamma }$
6s	0.5	− 0.003472	36.328316	0.101017	16.486441	− 6.52921	3.669771	9.957207
	1.0	− 0.0139	18.171143	0.201978	14.40713	− 4.449904	3.671383	9.957226
	2.0	− 0.056	9.080299	0.404376	12.327846	− 2.370805	3.671852	9.957041
	3.0	− 0.125	6.084213	0.603411	11.111565	− 1.154556	3.671281	9.957009
	4.0	− 0.222	4.541914	0.80906	10.248586	− 0.291635	3.674681	9.956951
7s	0.5	− 0.002551	49.576747	0.086018	17.3767	− 7.016642	4.264505	10.360058
	1.0	− 0.010204	24.790471	0.172067	15.297386	− 4.93739	4.265616	10.359996
	2.0	− 0.040816	12.388781	0.344528	13.218098	− 2.858331	4.268286	10.359767
	3.0	− 0.091837	8.255441	0.517394	12.001818	− 1.641522	4.271314	10.360296
	4.0	− 0.163265	6.191006	0.690437	11.138845	− 0.778891	4.2745	10.359954
8s	0.5	− 0.001953	64.581759	0.075439	18.149252	− 7.434644	4.871981	10.714608
	1.0	− 0.007812	32.273913	0.151015	16.06995	− 5.355824	4.873848	10.714126
	2.0	− 0.03125	16.137925	0.302288	13.99062	− 3.275737	4.878308	10.714883
	3.0	− 0.070312	10.752641	0.45413	12.774206	− 2.05662	4.883101	10.717586
	4.0	− 0.125	8.062925	0.60621	11.911413	− 1.197129	4.887825	10.714284
9s	0.5	− 0.001543	81.536051	0.067328	18.83181	− 7.803804	5.489621	11.028006
	1.0	− 0.006173	40.760914	0.134755	16.752463	− 5.725004	5.492741	11.027459
	2.0	− 0.024691	20.372147	0.269904	14.673131	− 3.64287	5.498518	11.030261
	3.0	− 0.056	13.582061	0.405377	13.45688	− 2.427931	5.505855	11.028949
	4.0	− 0.098765	10.183177	0.541329	12.593905	− 1.564951	5.512452	11.028954
10s	0.5	− 0.00125	100.515588	0.060827	19.443195	− 8.130654	6.1141	11.312541
	1.0	− 0.005	50.242804	0.121765	17.363776	− 6.050467	6.117807	11.313309
	2.0	− 0.02	25.113544	0.24398	15.284466	− 3.971259	6.127203	11.313207
	3.0	− 0.045	16.740561	0.366513	14.068172	− 2.754818	6.135626	11.313302
	4.0	− 0.08	12.552185	0.489656	13.205204	− 1.892796	6.146249	11.312408

**Table 2:** The analytical and numerical values of the variation (difference) of Shannon entropy in both position space and momentum space for cases of atomic number (*a*) $Z_1$, (*b*) $Z_2$.

			Analytical results		6s		7s		8s	
$Z_1$	$Z_2$	$X = \log _2 (Z_2 / Z_1)$	$\delta S_\rho (Z)$	$\delta S_\gamma (Z)$	$\delta S_\rho (Z)$	$\delta S_\gamma (Z)$	$\delta S_\rho (Z)$	$\delta S_\gamma (Z)$	$\delta S_\rho (Z)$	$\delta S_\gamma (Z)$
0.5	1.0	1.0	− 2.079442	2.079442	− 2.079311	2.079306	− 2.079314	2.079252	− 2.079302	2.07882
	2.0	2.0	− 4.158883	4.158883	− 4.158565	4.158405	− 4.158602	4.158311	− 4.158632	4.158907
	3.0	2.584963	− 5.375278	5.375278	− 5.374876	5.374654	− 5.374882	5.37512	− 5.375046	5.378024
	4.0	3.0	− 6.238325	6.238325	− 6.237855	6.237575	− 6.237855	6.237751	− 6.237839	6.237515
1.0	2.0	1.0	− 2.079442	2.079442	− 2.079284	2.079099	− 2.079288	2.079059	− 2.07933	2.080087
	3.0	1.584963	− 3.295837	3.295837	− 3.295565	3.295348	− 3.295568	3.295868	− 3.295744	3.299204
	4.0	2.0	− 4.158883	4.158883	− 4.158544	4.158269	− 4.158541	4.158499	− 4.158537	4.158695
2.0	3.0	0.584963	− 1.216395	1.216395	− 1.216281	1.216249	− 1.21628	1.216809	− 1.216414	1.219117
	4.0	1.0	− 2.079442	2.079442	− 2.07926	2.07917	− 2.079253	2.07944	− 2.079207	2.078608
3.0	4.0	0.415037	− 0.863046	0.863046	− 0.862979	0.862921	− 0.862973	0.862631	− 0.862793	0.859491

Now, using Eqs. (16) and (17), and logarithmic operation $\log _n M = \log _n b \times \log _b M$, the variation (difference) in $S_\rho$ and $S_\gamma$ for cases of the atomic number (*a*) $Z_1$, (*b*) $Z_2$ (holding *n* constant) can be written as

19 $$\begin{aligned} S_\rho (Z_2)-S_\rho (Z_1) = \delta S_\rho (Z) = -3X\ln 2 \approx -2.08X \end{aligned}$$

20 $$\begin{aligned} S_\gamma (Z_2)-S_\gamma (Z_1) = \delta S_\gamma (Z) = 3X\ln 2 \approx 2.08X \end{aligned}$$

where, $$X = \log _2 (Z_2 / Z_1)$$.

From Eqs. (19) and (20), we conclude that the variation of Shannon entropy with *Z* is independent to principal quantum number *n* and depends only to $Z_2 / Z_1$ and also observes that the variation of Shannon entropy in position space $S_\rho$ with *Z* is equal to negative of the variation of Shannon entropy in momentum space $S_\gamma$ with *Z* for constant *n* ($\delta S_\rho (Z) = -\delta S_\gamma (Z) \approx -2.08X$). Negative sign of $\delta S_\rho (Z)$ shows that $S_\rho$ decreases with increasing of *Z* and positive sign of $\delta S_\gamma (Z)$ show that $S_\gamma$ increases with increasing of *Z*. So, we can say that raising *Z* compresses the wavefunction in position space while expands wavefunction in momentum space in the same ratio. Due to this, total Shannon entropy $S_T$ remains constant with changing in *Z* for constant *n*. As a result, despite the compression of electron probability density, total Shannon entropy $S_T$ remains constant with a rise in *Z* for constant *n*, as Sen observed for the ground state Hydrogen-like atoms^85^. In Table 2, $Z_1$ and $Z_2$ are to indicate the atomic number, for example the quantities in Table are calculated separately for cases of atomic number (*a*) $Z=Z_1$, (*b*) $Z=Z_2$ and then their respective differences are evaluated. These results are verified by Eqs. (19) and (20) and equal value of $\frac{Z_2}{Z_1}$ have equal $\delta S_\rho (Z)$ and $\delta S_\gamma (Z)$ but opposite to each other for each principal quantum number *n*. A negative value of $\delta S_\rho (Z)$ shows that the wavefunction and electron probability density in position space is compressed (localized) with increases of *Z* and which compress in a ratio equal to $\delta S_\rho (Z)$, shown in Figs. 1 and 2. The wavefunction and electron probability density in momentum space expands (delocalizes) with increases in *Z* and expands in a ratio equal to $\delta S_\gamma (Z)$ if $\delta S_\gamma (Z)$ is positive.

The variation (difference) in $S_\rho$ and $S_\gamma$ for cases of principal quantum number (*a*) $n_1$, (*b*) $n_2$ (holding *Z* constant) can be written as

21 $$\begin{aligned} S_\rho (n_2)-S_\rho (n_1) = \delta S_\rho (n) \approx \frac{23}{4}N\ln 2 \approx 4.0N \end{aligned}$$

22 $$\begin{aligned} S_\gamma (n_2)-S_\gamma (n_1) = \delta S_\gamma (n) \approx -\frac{15}{4}N\ln 2 \approx -2.6N \end{aligned}$$

where, $$N = \log _2 (n_2 / n_1)$$.

From Eqs. (21) and (22), we conclude that the variation of Shannon entropy with principal quantum number *n* is independent to *Z* and depends only to $n_2 / n_1$ and also observe that the variation of Shannon entropy in position space $S_\rho$ with *n* is not equal to the variation of Shannon entropy in momentum space $S_\gamma$ with *n* for constant *Z* ($\delta S_\rho (n) \ne \delta S_\gamma (n)$). Increasing ’*n*’ results in the expansion of wavefunction in position space and compression of the wavefunction in momentum space but increase and decrease are not in the same ratio. Due to this, total Shannon entropy $S_T$ doesn’t remain constant with increase in *n* for constant *Z*. So, the variation of total Shannon entropy $S_T$ with *n* for constant *Z* as $\delta S_T (n) = \delta S_\rho (n) + \delta S_\gamma (n) \approx 1.4N$. Positive sign of $\delta S_\rho (n)$ shows that $S_\rho$ increases with increasing of *n* and negative sign of $\delta S_\gamma (n)$ show that $S_\gamma$ decreases with increasing of *n*.

For the free system, we can describe $S_\rho$ and $S_\gamma$ as functions of Energy *E* and principal quantum number *n*, respectively.

23 $$\begin{aligned} S_\rho (n,E) \approx \frac{11}{4}\ln n - \frac{3}{2}\ln 2 + \ln \pi - \frac{3}{2}\ln (-E) + 3 \end{aligned}$$

24 $$\begin{aligned} S_\gamma (n,E) \approx -\frac{3}{4}\ln n + \frac{13}{2}\ln 2 + 2\ln \pi + \frac{3}{2}\ln (-E) - \frac{10}{3} \end{aligned}$$

Summing Eqs. (23) and (24) and we get total Shannon entropy

25 $$\begin{aligned} S_T \approx 2\ln n + 5\ln 2 +3\ln \pi - \frac{1}{3}, \end{aligned}$$

Since total Shannon entropy, $S_T$ is independent of energy *E* for constant *n*. Now, using Eqs. (23) and (24) we can write the variation (difference) in $S_\rho$ and $S_\gamma$ for cases of Energy (*a*) $E_1$, (*b*) $E_2$ (holding *n* constant) as

26 $$\begin{aligned}S_\rho (E_2)-S_\rho (E_1) = \delta S_\rho (E) = -\frac{3}{2}Y\ln 2 \approx -1.04Y \end{aligned}$$

27 $$\begin{aligned}S_\gamma (E_2)-S_\gamma (E_1) = \delta S_\gamma (E) = \frac{3}{2}Y\ln 2 \approx 1.04Y \end{aligned}$$

where, $$Y = \log _2 (\frac{E_2}{E_1})$$.

From Eqs. (26) and (27), we conclude that the variation of Shannon entropy with *E* is also independent to principal quantum number *n* and depends only to $\frac{E_2}{E_1}$ and also observe that the variation of Shannon entropy in position space $S_\rho$ with *E* is equal to negative of the variation of Shannon entropy in momentum space $S_\gamma$ with *E* for constant *n* ($\delta S_\rho (E) = -\delta S_\gamma (E) \approx -1.04Y$). Negative sign of $\delta S_\rho (E)$ shows that $S_\rho$ decreases with increasing of $\frac{E_2}{E_1}$ and positive sign of $\delta S_\gamma (E)$ show that $S_\gamma$ increases with increasing of $\frac{E_2}{E_1}$.Due to this, total Shannon entropy $S_T$ remains constant with changing in energy *E* for constant principal quantum number *n*.

**Figure 3:**
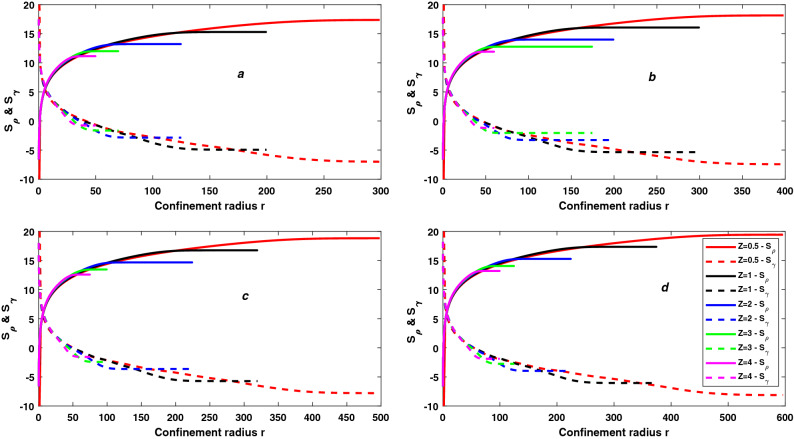
The variation of Shannon entropy in position space $S_\rho$ and Shannon entropy in momentum space $S_\gamma$ with confinement radius r and atomic number Z for 7s state (**a**), 8s state (**b**), 9s state (**c**) and 10s state (**d**) of trapped Hydrogen-like atoms.

**Figure 4:**
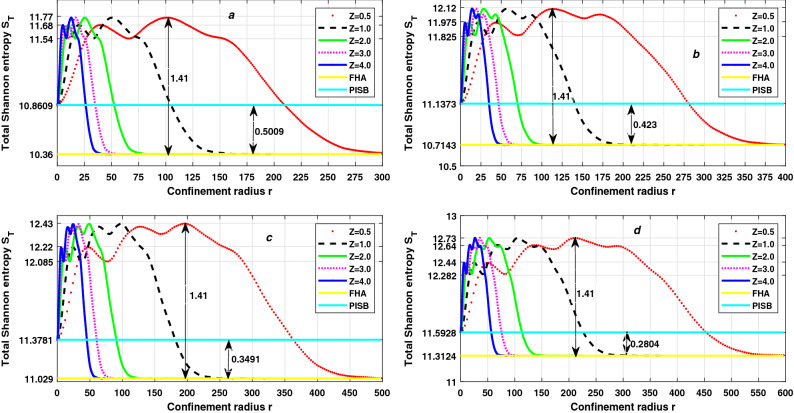
The variation of Total Shannon entropy $S_T$ with confinement radius r and atomic number Z for 7s state (**a**), 8s state (**b**), 9s state (**c**) and 10s state (**d**) of trapped Hydrogen-like atoms.

**Figure 5:**
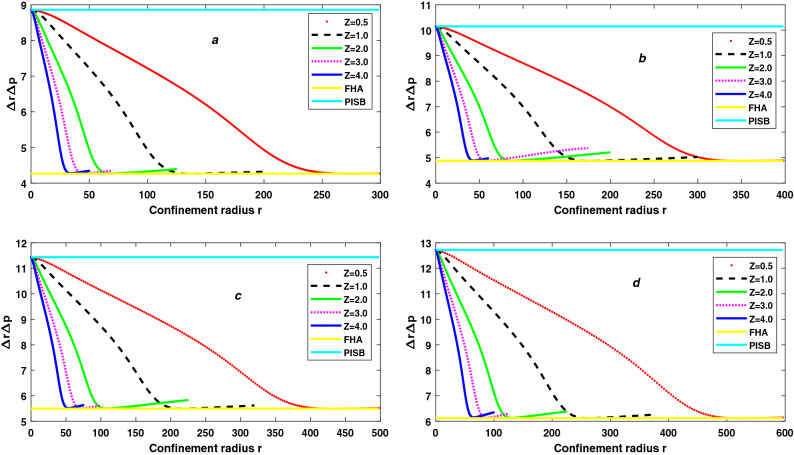
The variation of Heisenberg Uncertainty Principle $\Delta r\Delta p$ with confinement radius r and atomic number Z for 7s state (**a**), 8s state (**b**), 9s state (**c**) and 10s state (**d**) of trapped Hydrogen-like atoms.

### Trapped linear Rydberg states

There have been numerous studies on trapped Rydberg states. In Fig. 3, we have plotted the variation of Shannon entropy in position space $S_\rho$ and Shannon entropy momentum space $S_\gamma$ as a function of confinement radius for trapped linear Rydberg states of hydrogen-like atoms. As shown in insets, the results are for five values of atomic number namely *Z* = 0.5, 1, 2, 3, and 4. Fig. (3(a)) represents for 7*s* states, (3(b)) for 8*s* states, (3(c)) for 9*s* states and (3(d)) for 10*s* states. We observe that for all *s* states the value of Shannon entropy in position space $S_\rho$ decreases with increasing compression while Shannon entropy momentum space $S_\gamma$ increases, which is as expected. As a result, we can say that as compression increases, the spatial wavefunction becomes more localized and the momentum wavefunction becomes more delocalized. Except for free atom( at large value of *r* ), $\delta S_\rho / \delta r \ne -\delta S_\gamma / \delta r$ at each value of *r*. The Dirac-Fourier transform, which relates the densities in the position and momentum spaces differently under differently spatially confined spaces (different r), is the reason for this difference in the variation of two entropies^85^. Therefore, the total Shannon entropy $S_T = S_\rho + S_\gamma$ is not constant when *r* changes from the free atomic state ($r = \infty$) to 0. Total Shannon entropy $S_T$, corresponding to $S_\rho$ and $S_\gamma$ which are presented in Fig. 3, is displayed in Fig. 4. As in case of $S_\rho$ and $S_\gamma$, the results of $S_T$ are presented for five values of atomic number namely *Z* = 0.5, 1, 2, 3, and 4. The straight lines show the value of $S_T$ for two extreme cases, one for free Hydrogen atom (FHA) and second for particle in a spherical box (PISB). Figure (4(a)) represents for 7*s* states, (4(b)) for 8*s* states, (4(c)) for 9*s* states and (4(d)) for 10*s* states. We observe that the total Shannon entropy $S_T$ increases with increasing compression (*r* changes from $\infty$ to 0) and makes several peaks. Near tight confinement, $S_T$ decreases with increasing more compression and becomes the equal particle in a spherical box (PISB) for each value of *Z*. Because there is an equal number of nodes in the wavefunction for each value of atomic number *Z* for constant principal quantum number *n*, so, there is an equal number of peaks in the total Shannon entropy $S_T$ for each value of *Z* for constant principal quantum number *n*. But the position of each peak shifts to tight confinement with increasing the atomic number *Z* and it is inverse proportional to *Z* for constant *n* because the extension of the wavefunction decreases with the increasing the atomic number *Z* for constant principal quantum number *n*. But the height of each peak is independent of the atomic number *Z* for a constant principal quantum number *n*. The minimum value of the total Shannon entropy $S_T$ equals $S_T$ of the free hydrogen atom (FHA) for all values of *Z*. As a result, total Shannon entropy $S_T$ for free atoms is unaffected by the atomic number *Z*, as discussed in the previous subsection. The maximum value of the total Shannon entropy $S_T$ is the central peak which is nearest the critical radius ($r_c$). The critical radius is the characteristic radius of atoms, where the energy of atoms (E) becomes zero. And we have already discussed that height of the peak of $S_T$ is independent of the atomic number *Z* for constant principal quantum number *n*, so we can say the maximum total Shannon entropy $S_T$ is also independent of the atomic number *Z* for constant principal quantum number *n*. Also, there is an increasing number of nodes in wavefunction with increasing principal quantum number *n*. As a result, the number of peaks in the total Shannon entropy $S_T$ increases in perfect agreement with the principal quantum number *n*. From Figs. 1 and 4, it is interesting that the number of peaks in the total Shannon entropy $S_T$ is dependent on the number of nodes and they follow the relation

28 $$\begin{aligned} A= \frac{B}{2} = \frac{n - 1}{2} \end{aligned}$$

where, *A* = Number of peaks in $S_T$, *B* = Number of nodes in the wavefunction, and *n* principal quantum number.

So, we can say that the number of peaks in total Shannon entropy indicates the number of nodes in the wavefunction. But we know that extension of the wavefunction increases with increasing principal quantum number *n*. As a result, as the principal quantum number *n* increases, the position of each peak changes toward less confinement, while the atomic number *Z* remains constant. But the height of each peak increases with the increase of principal quantum number *n* for constant atomic number *Z*. So, the maximum total Shannon entropy $S_T$ increases with the increase of principal quantum number *n*. However, for free atoms, the total Shannon entropy $S_T$ is proportional to the principal quantum number *n*; as *n* increases, $S_T$ increases, as stated in the previous subsection. So, we can say that the minimum $S_T$ increases with increasing principal quantum number *n* in the same ratio of increase of the maximum $S_T$. Therefore, the difference between the maximum $S_T$ and the minimum $S_T$ is independent atomic number *Z* and principal quantum number *n*. From Fig. 4, the difference between maximum $S_T$ and minimum $S_T$ is always approximately 1.41 for confined linear states. However, the difference between the total Shannon entropy $S_T$ corresponding to a particle in a spherical box (PISB) and a free Hydrogen atom (FHA) decreases as the principal quantum number *n* increases. The fact that the kinetic energy of the electron in FHA varies as $\frac{1}{n^2}$, while the kinetic energy of the electron in PISB is directly proportional to $n^2$, provides a rough approximation of states where the difference in total Shannon entropy $S_T$ of PISB and FHA becomes almost zero. As a result, we expect these two entropies to equalize for $n\approx 15$ since the electron’s kinetic energy is the same in both FHA and PISB for $n\approx 15$. This behavior of the total Shannon entropy in the CHA, PISB(no coulombic potential), and FHA(no confinement) is due to the effects of the Coulomb potential/confinement^86^. In Figure 5, we have plotted the variation of the product of variance in position space $\Delta r$ and momentum space $\Delta p$ (Heisenberg’s Uncertainty) as a function of confinement radius for linear Rydberg states of confined hydrogen-like atoms. As shown in insets, the results are for five values of atomic number namely *Z* = 0.5, 1, 2, 3, and 4. The straight lines show the value of $\Delta r \Delta p$ for two extreme cases, one for free Hydrogen atom (FHA) and second for particle in a spherical box (PISB). Figure (5(a)) represents for 7*s* states, (5(b)) for 8*s* states, (5(c)) for 9*s* states and (5(d)) for 10*s* states. We observe that $\Delta r \Delta p$ decreases with decreasing compression (*r* changes from 0 to $\infty$). Near high compression (*r* $\rightarrow$ 0), $\Delta r \Delta p$ become the equal value of $\Delta r \Delta p$ of particle in a spherical box (PISB) for each value of *Z*. However, for free atom (*r* $\rightarrow$ $\infty$), $\Delta r \Delta p$ becomes equal to the value for free Hydrogen atom (FHA) for each value of *Z*. $\Delta r \Delta p$ increases as both the radius *r* and the atomic number *Z* of free atoms increase. So, Heisenberg’s Uncertainty $\Delta r \Delta p$ is not constant with the atomic number *Z* for increasing the radius of free Rydberg atoms. However, uncertainty based on Shannon entropy is independent of the atomic number *Z* for free atoms for increasing the radius of free Rydberg atoms. Also, the number of nodes in the wavefunction does not affect $\Delta r \Delta p$. Hence, uncertainty relation based on Shannon entropy is superior to Heisenberg uncertainty for trapped Rydberg atoms.

## Conclusion

In this work, the entropic measures like Shannon entropy in position and momentum spaces are presented for free and trapped Rydberg states of Hydrogen like atoms. We restricted our studies to principal quantum number ranging from 6 to 10. However, qualitative behaviour of these measures would reflect similar behaviour for higher excited states. We are able to compare the numerical values of Shannon entropy to analytical values for this system. We show that the total Shannon entropy $S_T$ in case of linear Rydberg states is independent of atomic number *Z* and of course it is depends on *n* (principal quantum number). It is worth to mention that we have been able to show the measure of contraction (expansion) of wavefunction in terms of the difference of the Shannon entropies in respective spaces in terms of *Z* (atomic number) and *n* (principal quantum number). To the best of our knowledge this dependence of wavefunction localization (delocalization) in quantitative manner has not been reported so far. It is also shown that Shannon entropic uncertainty are better measure compare to Heisenberg Uncertainty relation for trapped Rydberg atoms. Finally, we understand that work presented here will provide useful information for further studies in trapped Rydberg species.
